# Mutations in *OOEP* and *NLRP5* identified in infertile patients with early embryonic arrest

**DOI:** 10.1002/humu.24448

**Published:** 2022-08-30

**Authors:** Xiaomei Tong, Jiamin Jin, Zhanhong Hu, Yingyi Zhang, Heng‐Yu Fan, Yin‐Li Zhang, Songying Zhang

**Affiliations:** ^1^ Department of Obstetrics and Gynecology, Assisted Reproduction Unit, Sir Run Run Shaw Hospital Zhejiang University School of Medicine Hangzhou China; ^2^ Department of Obstetrics and Gynecology Key Laboratory of Reproductive Dysfunction Management of Zhejiang Province Hangzhou China; ^3^ Life Sciences Institute Zhejiang University Hangzhou China

**Keywords:** early embryonic arrest, female infertility, NLRP5, OOEP, SCMC

## Abstract

The subcortical maternal complex (SCMC), composed of several maternal‐effect genes, is vital for the development of oocytes and early embryos. Variants of SCMC‐encoding genes (*NLRP2*, *NLRP5*, *TLE6*, *PADI6*, and *KHDC3L*, but not *OOEP* and *ZBED3*) are associated with human oocyte maturation dysfunction, fertilization failure, and early embryonic arrest. In this study, we enrolled 118 Chinese patients who experienced recurrent preimplantation embryonic arrest during assisted reproductive technology treatments and performed whole‐exome sequencing. We discovered compound heterozygous missense variants (c.110G>C and c.109C>G) in the *OOEP* gene in one patient who experienced recurrent preimplantation embryonic arrest. Arrested embryos from this affected patient were analyzed by single‐cell RNA sequencing, which showed a downregulated transcriptome. In addition, six novel *NLRP5* variants (c.971T>A, c.3341T>C, c.1575_1576delAG, c.1830_1831delGT, c.1202C>T, and c.2378T>G) were identified in four patients with arrested and severely fragmented embryos. These suspicious mutations were examined by in vitro studies in HEK293T cells. Western blot analysis and immunofluorescence experiments showed that *OOEP* and partial *NLRP5* mutations caused decreased protein levels. Our findings first demonstrated that biallelic variants in *OOEP* gene could also cause human early embryonic arrest, similar to other SCMC components. We expanded the genetic mutation spectrum of SCMC genes related to early embryogenesis in humans, especially early embryonic arrest.

## 1 INTRODUCTION

Human infertility is influenced by various factors including exogenous and endogenous factors and the latter are typically genetic. Assisted reproductive technology (ART) is an efficient treatment for infertile couples without pathogenic variants. During this process, mature oocytes are fertilized with sperm by in vitro fertilization (IVF) or intracytoplasmic sperm (ICSI). However, some couples experience recurrent early embryonic arrest (EEA), preventing the development of viable embryos. The genetic determinants associated with nonviability in embryos largely remain unknown. Many RNAs and proteins are synthesized during the maturation of oocytes and are necessary for oocyte maturation, fertilization, and early embryo development. The genes encoding these functional maternal factors are named maternal‐effect genes. Recently, variants of maternal‐effect genes have been found to be associated with oocyte maturation, fertilization, and early embryogenesis in humans (L. Li et al., 2010; Lu et al., 2017).

The subcortical maternal complex (SCMC) is a multiprotein complex encoded by maternal‐effect genes and is essential for oocyte and early embryo development in mammalian species (L. Li et al., 2008; Zhu et al., 2015). The complex consists of many proteins, including transducin‐like enhancer of split 6 (TLE6), peptidyl arginine deiminase, type VI (PADI6), NLR family pyrin domain‐containing 5 (NLRP5), NLR family pyrin domain‐containing 2 (NLRP2), KH domain‐containing protein 3 (KHDC3L), oocyte expressed protein (OOEP), and zinc finger BED‐type containing 3 (ZBED3) in humans (Lu et al., 2017). In mice, knocking out any one of the SCMC‐coding genes causes infertility or subfertility due to EEA (Esposito et al., 2007; Gao et al., 2018; Mahadevan et al., 2017; Tong et al., 2000; Yu et al., 2014; P. Zheng & Dean, 2009). In humans, some SCMC gene mutations have been reported to be associated with oocyte maturation defects, fertilization failure, or embryonic arrest at the cleavage stage. Biallelic mutations in *TLE6* (OMIM #611689) lead to early embryonic developmental disorders for the first time in three consanguineous families (Alazami et al., 2015). Subsequently, mutations in *PADI6*, *KHDC3L*, *NLRP5*, and *NLRP2* genes were found in patients with repeated ART failure, leading to oocyte maturation disorder and EEA at the cleavage stage (Maddirevula et al., 2017; Mu et al., 2019; Wang et al., 2018; Xu et al., 2016). However, *OOEP* and *ZBED3* gene mutations have not been reported to be associated with early human embryonic development.

In this study, we enrolled typical patients who experienced more than two rounds of EEA during IVF/ICSI treatments without specific reasons. Whole‐exon sequencing was used to identify rare variants in SCMC genes. *OOEP* mutations were identified in EEA patients for the first time, and we also found six novel heterozygous mutations in *NLRP5* in four patients from three families. We investigated the disruptive effects of these mutations through single‐cell RNA‐seq of abandoned embryos. In vitro studies were also conducted to investigate the potential underlying molecular mechanisms. We believe these mutations may contribute to EEA in infertile individuals.

## 2 MATERIALS AND METHODS

### 2.1 Clinical samples

A total of 118 infertile patients who experienced more than two ART treatment failures due to EEA were recruited from the Sir Run Run Shaw Hospital affiliated with Zhejiang University School of Medicine. After informed consent was obtained from participants, peripheral blood samples were collected, and genomic DNA was extracted. Arrested embryos were donated by the patients. Control 0PN control embryos were obtained from donors with male factor infertility who underwent successful IVF/ICSI attempts. This study was approved by the ethical committee of Sir Run Run Shaw Hospital affiliated with Zhejiang University School of Medicine (no. Y21H040019).

### 2.2 Genetic studies

Whole‐exome capture was performed using the SeqCap EZ Human Exome Kit (Roche). Illumina HiSeq. 3000 platform (Illumina) was used for sequencing. Sequencing results were compared with the human reference sequence (NCBI Genome build GRCh37). Variants were annotated with the GRCh37, dbSNP (version 138). Homozygosity mapping was performed with Homozygosity Mapper. The candidate variants met the following criteria: (1) the frequencies of compound heterozygous or homozygous variants were less than 0.1% in the Genome Aggregation Database (GnomAD) databases (Lek et al., 2016), (2) variants were predicted to be loss‐of‐function or damaging by Combined Annotation Dependent Depletion (CADD; GRCh37‐v1.6 version, score ≥ 20) (Rentzsch et al., 2021) and Protein Variation Effect Analyzer (PROVEAN, v1.1.3 version, score ≤ −2.5) (Choi et al., 2012), and (3) variant related genes were associated with early embryo development. Candidate variants were evaluated according to the American College of Medical Genetics and Genomics and the Association for Molecular Pathology (ACMG/AMP) guidelines (Richards et al., 2015). Detailed whole‐exome sequencing data are shown in Supporting Information: Table S1.

Candidate variants were confirmed by Sanger sequencing. Genomic DNA from peripheral blood was isolated using the MiniBEST Universal Genomic DNA Extraction Kit, 9756 (TaKaRa). KAPA HiFi HotStart ReadyMix (kk2602; Kapa Biosystems) was used for PCR, and an ABI 3100 DNA analyzer (Applied Biosystems) was applied for Sanger analysis. The primers used for the Sanger analysis are shown in Supporting Information: Table S2.

### 2.3 Expression vector construction and mutagenesis

Full‐length coding sequences of the *NLRP5* and *OOEP* genes were amplified from oocyte complementary DNA (cDNA) from healthy women and cloned into the PDEST‐Entry vector with a FLAG‐tag and human influenza hemagglutinin (HA)‐tag. Site‐directed mutagenesis was performed to build mutant plasmids using a QuickChange II Site‐Directed Mutagenesis Kit (200523; Agilent). Wild‐type and mutant plasmids were confirmed by Sanger sequencing. The primers used for the mutagenesis are listed in Supporting Information: Table S3.

### 2.4 Western blot analysis

OOEP and NLRP5 wild‐type and mutant plasmids were transfected into HEK 293T cells using Lipo3000 In Vitro DNA Transfection Reagent (L3000015; Thermo Fisher Scientific) according to the manufacturer's instructions. Lysed proteins were separated on a 12.5% sodium dodecyl sulfate–polyacrylamide gel electrophoresis (SDS‐PAGE) gel and transferred onto polyvinylidene difluoride (PVDF) membranes (Millipore Corp.). Anti‐FLAG antibody (14793, dilution: 1:1000; Cell Signaling Technology), anti‐HA antibody (3724, dilution: 1:1000; Cell Signaling Technology), and anti‐β‐actin antibody (K200058M, dilution: 1:2000; Solarbio) were the primary antibodies. Secondary antibodies goat‐anti‐mouse immunoglobulin G (IgG) and goat‐anti‐rabbit IgG (7074 and 7076, dilution: 1:5000; Cell Signaling Technology) were subsequently applied. Finally, the membranes were visualized by enhanced chemiluminescence (WBKLS0500; Millipore Corp.).

### 2.5 Immunofluorescence

HEK 293T cells transfected with OOEP or NLRP5 wild‐type and mutant plasmids were fixed in 4% paraformaldehyde. The cells were incubated with an anti‐HA antibody (3724, dilution: 1:1000; Cell Signaling Technology) or an anti‐FLAG antibody (14793, dilution: 1:1000; Cell Signaling Technology) at 4°C overnight. Then, HEK 293T cells were incubated with Alexa Fluor 488 and 568 goat anti‐rabbit secondary antibodies (A32731 and A11036; Thermo Fisher Scientific) at a 1:200 dilution for 1 h. The nuclei were stained with DAPI (4′,6‐diamidino‐2‐phenylindole) (D1306; Thermo Fisher Scientific) at a 1:200 dilution. All the images were captured on a confocal laser‐scanning microscope (LSM800; Zeiss).

### 2.6 Single‐cell embryo RNA sequencing (RNA‐seq)

Three 8‐cell embryos developed from 0PN zygotes were donated from control patients. Three arrested 4‐cell embryos were acquired from the patient with compound heterozygous *OOEP* mutation. Single embryo RNA‐seq was performed as previously described (Y. L. Zhang et al., 2021). Briefly, each embryo was lysed directly in the lysis buffer, and cDNA was synthesized via SMARTScribe™ Reverse Transcriptase (Clontech). The SMART‐Seq v4 Ultra Low Input RNA Kit (Clontech) was used to prepare the low‐input library. Afterward, sequencing was performed on Illumina platforms using a 2 × 150‐bp paired‐end‐sequencing protocol.

Cutadapt software (cutadapt‐1.9) was used to remove adapter contamination reads. HISAT2 software was used (hisat2‐2.0.4) to map reads to the *Homo sapiens* Ensembl v96 genome. The mapped reads were assembled using StringTie (stringtie‐1.3.4d). The expression levels of all transcripts were estimated and used to calculate FPKM. The mRNAs selected with fold change > 2 or fold change < 0.5 and *p* < 0.05 by edgeR were considered differentially expressed. The Gene Ontology (GO) terms and Kyoto Encyclopedia of Genes and Genomes pathways of these differentially expressed mRNAs were also annotated.

## 3 RESULTS

### 3.1 Clinical characteristics of the affected individuals

Five patients in this study were from four independent families and all experienced infertility without an explicit etiology for over 2 years. Their husbands' sperm displayed normal count, morphology, and motility characteristics.

**Figure 1:**
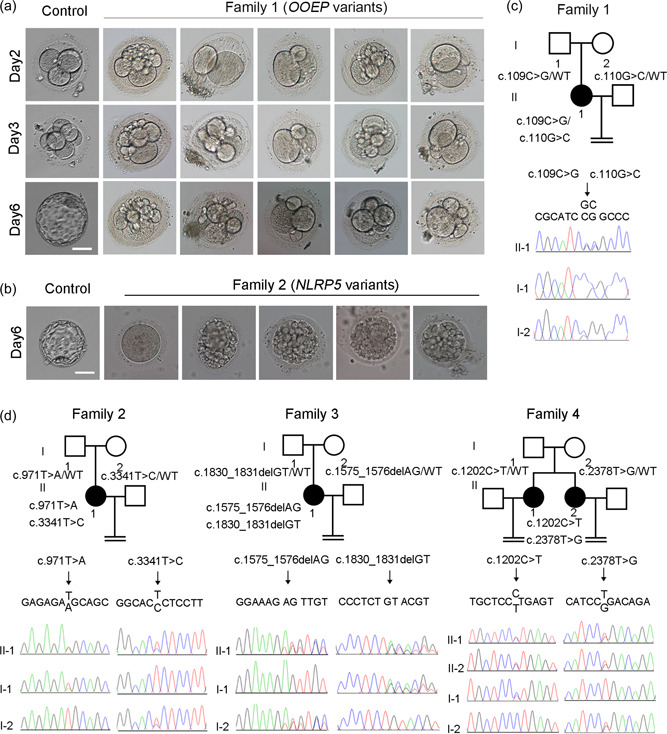
Identification of mutations in *OOEP* and ***NLRP5***. (a) The morphologies of control embryos and embryos from proband II‐1 in family 1 carrying *OOEP* mutations were examined by light microscopy on Days 2, 3, and 6 after fertilization. Scale bar = 40 μm. (b) The morphologies of control embryos and embryos from proband II‐1 (Family 2) were examined by light microscopy on Day 6 after fertilization. Scale bar = 40 μm. (c) Pedigrees of the family carrying *OOEP* mutations. Sanger sequencing confirmation is shown below the pedigrees. (d) Pedigrees of three families carrying *NLRP5* mutations. Sanger sequencing confirmation is shown below the pedigrees. Squares denote male family members, circles denote female members, solid black circles denote probands, and the equals sign denotes infertility.

Patient 1 in family 1 was 25 years old at the time of examination with a 2‐year history of primary infertility. She underwent two IVF attempts at a different hospital, two IVF attempts and one ICSI attempt at our hospital. A total of 56 oocytes were retrieved with a 75% maturation rate (42/56). Twenty‐six of the MII oocytes (61.9%) were normally fertilized with two pronuclei (2PN). Twenty‐four of them were cleaved, and all embryos were arrested at the two to six‐cell stage on Day 3 with a fragmentation rate of 10%–80% (Table 1). The morphology of the arrested and fragmented embryos from patient 1 and control patients was captured on Days 2, 3, and 6 (Figure 1a). Compared with the continuous proliferation and formation of a blastocyst, abnormal divisions were observed in patient 1, mostly presenting as delayed cell division from Day 2, eventually leading to scattered fragments (Figure 1a).

**Table 1:** Clinical characteristics of patients with *OOEP* and *NLRP5* mutations

Gene	Case	Age (years)	Duration of infertility (years)	IVF/ICSI cycles	Total oocytes	MII oocytes	Normal fertilized oocytes	Cleavage embryos	Viable embryos on Day 3	Embryo outcomes
*OOEP*	1	25	2	1st IVF	13	11	5	5	0	Arrested during subsequent blastocyst culture^b^
		25	2	2nd IVF	7	7	7	5	0	Arrested during subsequent blastocyst culture^b^
		26	3	3rd IVF	13	11	5	5	0	3 × 2‐cell, 2 × 4‐cell, arrested with 25%–60% fragmentation. Two 4‐cell embryos were further cultured but failed to form blastocysts on Day 5
		27	4	4th ICSI	17	8	4	4	0	2 × 2‐cell, 2 × 3‐cell, arrested with 40%–80% fragmentation
		27	4	5th IVF	6	5	5	5	0	2 × 2‐cell, 1 × 3‐cell, 1*4‐cell, 1 × 6‐cell arrested with 10%–30% fragmentation. These embryos were further cultured but failed to form blastocysts on Day 5
*NLRP5*	2	23	5	1st IVF	21	17	11	5	0	4 × 2‐cell, 1 × 3‐cell arrested with 70%–90% fragmentation
		23	5	2nd IVF	27	24	11	7	0	5 × 2‐cell, 2 × 3‐cell, arrested with 50%–80% fragmentation
		24	6	3rd IVF	11	10	5	1	0	1 × 2‐cell, arrested with 80% fragmentation
		24	6	4th ICSI	4	4	2	1	0	1 × 2‐cell, arrested with 70% fragmentation
	3	31	2	1st ICSI	4	4	2	0	0	Cleavage arrest
		32	3	2nd ICSI^a^	12	12	5	1	0	1 × 2‐cell, arrested with 80% fragmentation
		33	4	3rd ICSI	6	5	0	0	0	Fertilization failure
	4	31	8	1st IVF	10	10	4	4	0	1 × 2‐cell, 1 × 4‐cell, 1 × 6‐cell, and 1 × 8‐cell, arrested during subsequent blastocyst culture
		32	9	2nd IVF	14	14	13	6	3	1 × 2‐cell, 1 × 4‐cell, 1 × 6‐cell, 3 × 8‐cell, implantation failure after 8‐cell transfer, others arrested^b^
		38	15	3rd ICSI	12	7	4	2	0	1 × 2‐cell, 1 × 4‐cell, arrested with 40%‐80% fragmentation
		38	15	4th ICSI	9	8	7	7	0	6 × 2‐cell, 1 × 3‐cell, arrested with 60%–80% fragmentation
	5	43	10	1st ICSI	7	7	6	6	0	1 × 2‐cell, 2 × 3‐cell, 2 × 4‐cell, 1 × 5‐cell arrested with 10%–70% fragmentation. Three embryos at 4–5‐cell stage were further cultured but failed to form blastocysts on Day 5

Abbreviations: ICSI, intracytoplasmic sperm injection; IVF, in vitro fertilization; MII, metaphase II.

^a^ Half of MII oocytes were assisted with artificial oocyte activation.

^b^ The attempts conducted at other hospitals without a more detailed description of the arrested embryos.

The second patient from family 2 was 23 years old at examination and exhibited primary infertility for 5 years. She had undergone three IVF attempts and one ICSI attempt, all of which failed. A total of 63 oocytes were retrieved; 55 were mature (87.3%) with a normal fertilization rate (52.7%), and 14 of 29 2PN zygotes were cleaved at the 2‐ to 3‐cell stage on Day 3. However, these early embryos were all arrested, with a fragmentation rate of 50%–80% after Day 3 (Table 1). On Day 6, embryos from control patients formed a blastocyst, whereas the embryos of patient 2 presented with arrested development and severe fragmentation (Figure 1b).

Patient 3 from family 3 was 31 years old at the time of examination with a 2‐year history of primary infertility. She underwent three failed ICSI attempts. A total of 22 oocytes were retrieved; 21 were mature (95.5%), of which seven were normally fertilized (33.3%), six were arrested at the zygote stage, and the remaining one was arrested at the 2‐cell stage on Day 3 (Table 1).

Patients 4 and 5 were sisters from family 4. Patient 4 underwent two IVF attempts at a different hospital and two ICSI attempts at our hospital. The previous two IVF attempts were carried out when the patient was 31 years old; 24 oocytes were retrieved, and 17 (70.8%) were normally fertilized. On Day 3, 10 of the 17 2PN zygotes developed into two 2‐cell embryos, two 4‐cell embryos, two 6‐cell embryos, and four 8‐cell embryos. Three 8‐cell embryos were transferred twice, but the patient did not become pregnant. The remaining seven embryos were arrested during subsequent blastocyst culture (Table 1). After two failed IVF attempts, the patient achieved natural conception at the age of 33 but subsequently experienced a spontaneous abortion. The next two ICSI attempts were carried out when the patient was 38 years old. Fifteen out of 21 (71.4%) retrieved oocytes were mature, of which 11 (73.3%) were normally fertilized. Nine 2PN zygotes were cleaved and arrested at the 2‐cell to 4‐cell stage on Day 3 with a fragmentation rate of 40%–80% (Table 1). Patient 5 also had a history of spontaneous abortion at the age of 33. She had undergone one failed rescue ICSI attempt at the age of 43. Seven retrieved oocytes were mature (100%), of which six (85.7%) were normally fertilized and underwent cleavage; however, all embryos were arrested at the 2‐cell to 5‐cell stage on Day 3 with a fragmentation rate of 10%–70% (Table 1).

### 3.2 Genetic identification of *OOEP* and *NLRP5* mutations

Through whole‐exome sequencing, we identified that patient 1 carried compound heterozygous *OOEP* (RefSeq NM_001080507.3) mutations and patients 2–5 carried compound heterozygous *NLRP5* (RefSeq NM_153447.4) mutations. Detailed whole‐exome sequencing information is shown in Supporting Information: Table S1. Sanger sequencing confirmed that all of the patients had compound heterozygous mutations, and the mutations were inherited from their parents in a recessive pattern in all four families (Figures 1c,d).

**Figure 2:**
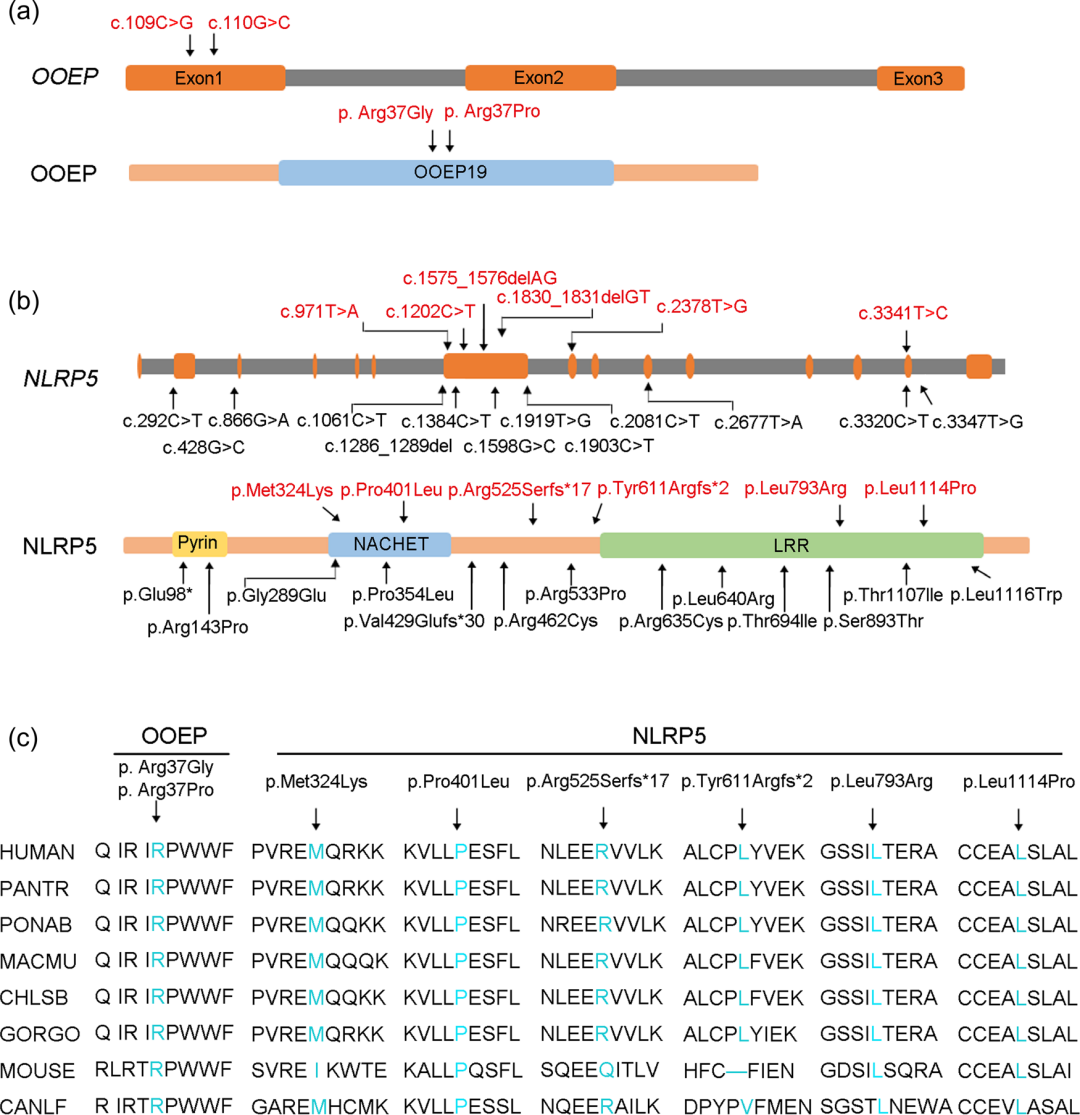
Locations and conservation of mutations in *OOEP* and *NLRP5* proteins. (a) The positions of two novel mutations are indicated in the gene and amino acid structure of OOEP. (b) The locations of all mutations are indicated in the gene and amino acid structure of NLRP5. The six novel mutations are marked in red and the reported mutations are represented in black in the schematic diagram. (c) The affected amino acids in OOEP and NLRP5 were compared among eight mammalian species in a conservation analysis.

Patient 1 had two missense mutations in *OOEP*, c.109C>G (p.Arg37Gly) and c.110G>C (p.Arg37Pro). The two missense mutations induced the alteration of the same amino acids (Arg 37) and were inherited from her parents. *OOEP* contains three exons and encodes a 149 amino acid protein. Arg 37 is localized in the N‐terminus of OOEP and is conserved among many mammalian species (Figure 2a,b). The allele frequencies of c.109C>G and c.110G>C in *OOEP* were not available in the GnomAD database. The two variants in *OOEP* were predicted as damaging according to CADD software (Table 2).

**Table 2:** Overview of the *OOEP* and NLRP5 mutations observed in the four families

GENE	Observed in families	Genomic position (bp)	Exon	cDNA change‐HGVS	Protein change	Transcripts	CADD^a^	PROVEAN^a^	GnomAD^b^	ACMG
*OOEP*	Family 1	Chr 6: 74079407	1	c.109C>G	p.Arg37Gly	NM_001080507.3	D	D	NA	VUS
		Chr 6: 74079406	1	c.110G>C	p.Arg37Pro		D	T	NA	VUS
*NLRP5*	Family 2	Chr 19: 56538570	7	c.971T>A	p.Met324Lys	NM_153447.4	T	D	NA	VUS
		Chr 19: 56569647	14	c.3341T>C	p.Leu1114Pro		D	D	NA	VUS
	Family 3	Chr 19: 56539171	7	c.1575_1576delAG	p.Arg525Serfs*17		NA	NA	NA	LP
		Chr 19: 56539427	7	c.1830_1831delGT	p.Tyr611Argfs*2		NA	NA	NA	LP
	Family 4	Chr 19: 56538801	7	c.1202C>T	p.Pro401Leu		D	D	NA	VUS
		Chr 19: 56544078	8	c.2378T>G	p.Leu793Arg		D	D	NA	VUS

Abbreviations: cDNA, complementary DNA; D, damaging; LP, Likely pathogenic; NA, not available; T, tolerable; VUS, Variant Uncertain Significance.

^a^ Mutation assessment by Combined Annotation Dependent Depletion (CADD) and Protein Variation Effect Analyzer (PROVEAN).

^b^ Frequency of corresponding mutations in the Genome Aggregation Database (GnomAD) in all populations.

Patient 2 had two missense mutations in *NLRP5*, c.971T>A (p.Met324Lys) and c.3341T>C (p.Leu1114Pro). Patient 3 carried two frameshift mutations in *NLRP5*, c.1575_1576delAG (p.Arg525Serfs*17) and c.1830_1831delGT (p.Tyr611Argfs*2). Patient 4 and patient 5 in family 3 had the same two missense mutations in *NLRP5*, c.1202C>T (p.Pro401Leu) and c.2378T>G (p.Leu793Arg). Each patient's mutations were inherited from heterozygous parents, indicating a biallelic mutation in affected individuals. These six mutation sites are labeled on the schematic map of the *NLRP5* gene (red labeled, Figure 2b) and have not been previously reported. As shown in Figure 2c, the amino acids altered by these mutation sites were highly conserved, as shown in the alignment of NLRP5 proteins across different species. The allele frequencies of the six mutations in *NLRP5* were not found in GnomAD database (Table 2). The six variants in *NLRP*5 were predicted as damaging according to PROVEAN (Table 2). Two frameshift mutations in *NLRP5*, c.1575_1576delAG and c.1830_1831delGT were considered likely pathogenic, and other variants of *NLRP5* and *OOEP* were considered as variant uncertain significance by ACMG. Collectively, these results indicate that biallelic mutations in *OOEP* and *NLRP*5 may be a potential genetic cause of female infertility.

### 3.3 Expression and localization of the mutant OOEP and NLRP5 proteins in HEK 293T cells

**Figure 3:**
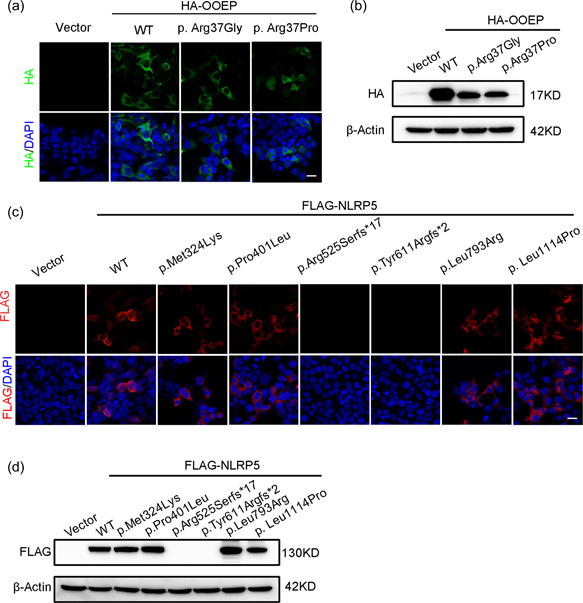
Subcellular localization and western blot analysis of the wild‐type and mutant OOEP and NLRP5 proteins. (a) The effects of the mutations on OOEP protein levels determined by immunofluorescence analysis of HEK293T cells transfected with wild‐type or mutant vectors. Scale bar = 20 μm. (b) The effects of the mutations on OOEP protein levels determined by western blot analysis analysis of HEK293T cells transfected with wild‐type or mutant vectors. (c) The effects of the mutations on NLRP5 protein levels determined by immunofluorescence analysis of HEK293T cells transfected with wild‐type or mutant vectors. Scale bar = 20 μm. (d) The effects of the mutations on NLRP5 protein levels determined by western blot analysis analysis of HEK293T cells transfected with wild‐type or mutant vectors.

Since it is unknown whether mutations of *OOEP* are associated with human infertility, wild‐type and mutant *OOEP* vectors were transfected into HEK 293T cells to evaluate the functional effects of the identified OOEP mutations in vitro. The c.109C>G (p.Arg37Gly) and c.110G>C (p.Arg37Pro) mutations reduced the expression of the OOEP protein compared with that in the wild type, as shown by immunofluorescence (Figure 3a) and western blot analysis (Figure 3b). These results indicate that mutations in *OOEP* impair the stability of the OOEP protein in HEK 293T cells.

Next, we evaluated the effects of the six novel mutations in *NLRP5* on protein properties. Similarly, wild‐type and mutant NLRP5 plasmids were also transfected into HEK 293T cells. The p.Arg525Serfs*17 and p.Tyr611Argfs*2 mutations caused a dramatic loss of NLRP5 protein expression compared with that in the wild type, as shown by immunofluorescence (Figure 3c) and western blot analysis (Figure 3d). The c.971T>A (p.Met324Lys), c.3341T>C (p.Leu1114Pro), c.1202C>T (p.Pro401Leu) and c.2378T>G (p.Leu793Arg) mutations did not cause statistically significant reductions in NLRP5 protein expression levels. These results indicate that the two frameshift mutations in NLRP5 cause protein instability, while the missense mutations do not induce dramatic changes in protein expression levels.

### 3.4 Lack of OOEP modestly affected zygotic genome activation in human early embryos

**Figure 4:**
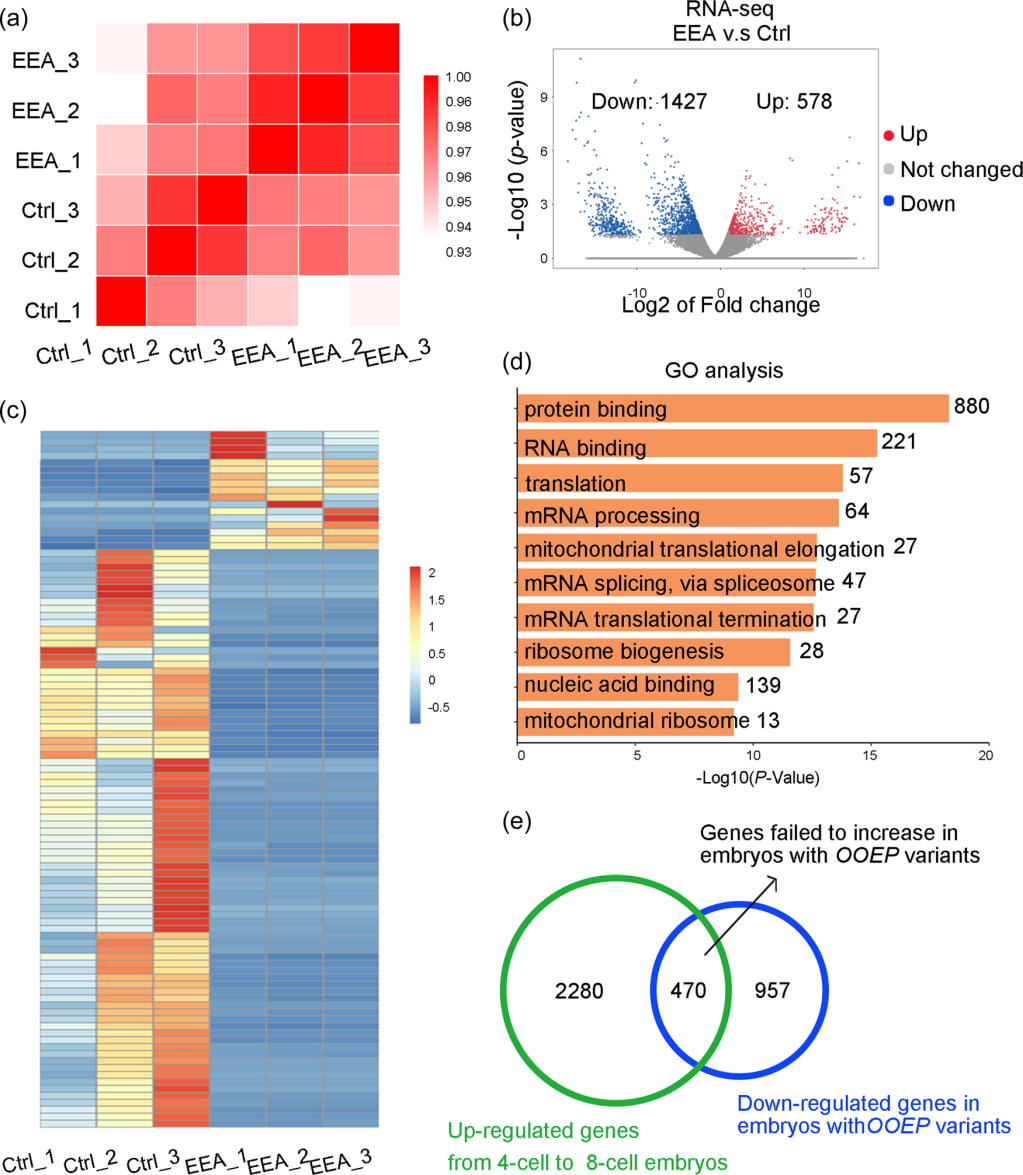
Single‐cell RNA‐seq analysis of arrested embryos from patients with *OOEP* variants. (a) The Pearson correlation analysis of RNA‐seq results of embryos acquired from control patients and patients with *OOEP* mutations. (b) Volcano plots showing transcriptional changes between control and patient embryos. Genes with decreases or increases of more than 0.5‐ or 2‐fold are indicated in blue and red, respectively. (c) Heatmap showing the expression dynamics of embryos from affected and control patients. (d) Enriched biological process of downregulated genes in embryos affected by *OOEP* variants, obtained via Gene Ontology (GO) analysis. (e) Venn diagram showing the downregulated genes in embryos affected by *OOEP* variants overlapping with the upregulated genes at the normal 4‐cell to 8‐cell transition. EEA, early embryonic arrest; RPKM, reads per kilobase per million mapped reads.

To explore the potential effects of *OOEP* mutations on the transcriptome of early embryo development, we performed RNA‐seq using abandoned embryos from control individuals and from the patient carrying *OOEP* compound heterozygous variants. Pearson correlation analysis showed that the mRNA profiles of replicates of the two groups were comparable (Figure 4a). In the mutant *OOEP*‐affected embryos, compared with the control embryos, 1427 gene transcripts were downregulated, and 578 transcripts were upregulated with a threshold of 0.5‐ and 2‐fold, respectively (Figures 4b and 4c). This result indicates that a substantial number of mRNAs were not transcribed or were decayed in the affected embryo, which may result from mutant *OOEP* protein expression. The GO analysis results revealed that these downregulated genes were mainly enriched in protein binding, translation, mRNA processing, and mitochondrial function (Figure 4d). According to Yan's published data (Yan et al., 2013), the most substantial changes or zygotic genome activation (ZGA) occur during the 4‐cell to 8‐cell transition, during which the expression of 2750 genes increases (RPKM > 0.5 and fold change > 2). Among the 1427 downregulated transcripts in *OOEP* mutant embryos, 470 overlapped with genes with substantially increased expression in 8‐cell embryos (Figure 4e). This result indicates that *OOEP* mutation modestly affected ZGA in human embryos. In conclusion, *OOEP* mutations hamper the transcription of some genes that may be essential for early embryonic development.

## 4 DISCUSSION

In this study, we identified rare and novel variants in *OOEP* and *NLRP5*. We found that two missense mutations in *OOEP* (c.110G>C and c.109C>G) impair the stability of the OOEP protein in vitro. This is the first time that mutations in *OOEP* have been found to be associated with EEA in humans. Six novel *NLRP5* gene mutations were also reported. Two frameshift mutations (c.1575_1576delAG and c.1830_1831delGT) impaired the stability of the NLRP5 protein in HEK 293T cells.

The SCMC is a conserved multiprotein module of the mammalian oocyte that orchestrates several essential events during the oocyte‐to‐embryo transition, such as F‐actin dynamics, mitochondrial organization, genome stability, and symmetric cell division in early embryos (Lu et al., 2017). Several studies have demonstrated that mutations in SCMC‐related genes (*TLE6*, *PADI6*, *NLRP2*, *NLRP5*, and *KHDC3L*) cause human female infertility in the form of an exhibition of EEA and fragmentation (Maddirevula et al., 2017; Mu et al., 2019; Wang et al., 2018; Xu et al., 2016). Mutations in *OOEP* and *ZBED3*, components of the SCMC, have not previously been associated with human female infertility. In addition to gene mutations involving the SCMC, our group, and Wang's group found that biallelic mutations in the *MOS* gene cause EEA and female infertility (Zeng et al., 2022; Y. L. Zhang et al., 2022). In the five patients from four independent families, we confirmed that none of the five patients carried *MOS* mutations through whole‐exome sequencing analysis.

Cell division defects have been reported in Moep (the murine homolog of OOEP)‐ablated mouse embryos, causing 2‐cell to 4‐cell stage arrest (Tashiro et al., 2010). Consistently, in our study, the affected patient with compound heterozygous *OOEP* gene mutations showed normal oocyte maturation and fertilization rates. However, the embryos were all arrested, mainly at the 2‐cell to 4‐cell stage on Day 3. According to our in vitro experiments, the *OOEP* gene mutation reduced OOEP protein levels. We believe that unstable OOEP proteins resulting from *OOEP* mutations may induce SCMC dysfunction, eventually leading to early embryonic development failure.

Meanwhile, our RNA‐seq showed that embryos carrying *OOEP* mutations failed to activate 470 (17% of 2750 ZGA genes) gene transcription between the 4‐cell and 8‐cell stages when the major maternal‐zygotic transition occurs. It has been proven that the SCMC is required for many biological processes during the oocyte‐to‐embryo transition, including zygotic genome activation (Lu et al., 2017), because a lack of *Nlrp5* or *Padi6* in mice partially affects zygotic genome transcription at the 2‐cell stage (Tong et al., 2000; Yurttas et al., 2008). However, the effect of the SCMC on ZGA has not been reported in human embryos. Our results first demonstrated that *OOEP* mutation modestly affected ZGA in early human embryos. Since OOEP is mainly localized in oocytes and the cellular cytoplasm, we suggest that this slight change in ZGA may be the secondary effect of the loss of OOEP.

In addition, in a previous report, the child of a mother carrying the c.109C>T homozygous mutation in *OOEP* was diagnosed with multilocus imprinting disturbances (MLIDs) (Begemann et al., 2018). MLIDs are caused by DNA methylation disturbance of multiple imprinted genes across the genome and show varying clinical symptoms. Because of the limited information available, the relationship between the *OOEP* mutation and the child's phenotype could not be confirmed. Moreover, the patient in our study who carried the c.109C>G and c.110G>C mutations experienced infertility with EEA. The reasons why similar genotypes cause different phenotypes need to be further explored.

*Nlrp5* (Mater) was the first maternal‐effect gene found in mice in which maternal ablation caused EEA at the 2‐cell stage (Tong et al., 2000). Mu et al. (2019) first reported three individuals from two families carrying biallelic mutations in *NLRP5* that contributed to human EEA in 2019. To date, five studies have focused on 13 variants (Figure 2b) and demonstrated that *NLRP5* mutations are responsible for oocyte maturation dysfunction, fertility disorder, and EEA (Huang et al., 2022; M. Li et al., 2021; Mu et al., 2019; Xu et al., 2020; W. Zheng et al., 2021). Consistent results were observed in the five patients in our study. All patients with *NLRP5* mutations had a normal oocyte maturation rate (71.4%–100%). Patient 3 had frameshift mutations in NLRP5, exhibiting a decreased fertilization rate (33.3%). In addition, the affected women with missense variants (patients 2, 4, and 5) had a limited number of arrested 2‐cell to 8‐cell embryos on Day 3. The 8‐cell embryos were transferred or cultured for blastocysts without success. Patient 3, with compound heterozygous frameshift mutations in *NLRP5*, had a more severe phenotype than the other three patients with compound missense mutations (patients 2, 4, and 5). In addition to the low fertilization rate in patient 3, most fertilized oocytes were arrested at the zygote stage. Only one 2‐cell embryo was derived but failed to further develop. Our studies further confirmed that NLRP5 is essential for human fertilization and early embryo cleavage.

Interestingly, in family 4, both sisters who carried compound heterozygous missense mutations presented with difficulty in conceiving and had a history of spontaneous abortion. Studies have revealed that women carrying *NLRP5* or other maternal‐effect gene variants experience pregnancy loss or have offspring with MLIDs (Begemann et al., 2018; Docherty et al., 2015; Hui et al., 2017). However, imprinting disorders were not analyzed in the presented cases. The clinical features of patients with mutations of the same maternal gene were different, suggesting that different types of mutations and other modified genes together with environmental factors affect the clinical penetrance. Here, we suggest that residual NLRP5 proteins resulting from missense mutations may have a reduced ability to stabilize the SCMC, which supports embryo development to some extent. However, with increasing age, unstable SCMCs are more vulnerable and unable to support embryo development after Day 3. Studies of sheep oogenesis also revealed that developing oocytes in aged animals had decreased SCMC mRNA levels (Bebbere et al., 2020). The combination of SCMC gene mutations and the age‐related decline in SCMC mRNA may work together to diminish embryonic developmental potential. However, more studies on advanced age and SCMC stability should be conducted.

There are some limitations to our study. First, the missense variants of *NLRP5* showed no significant effect on the protein level. Although there was no allele frequency in the databases and the variants were predicted to be damaging or possibly damaging, the exact effect of these missense variants on NLRP proteins remains to be determined. Second, we only found one patient who carried *OOEP* mutations in a small group of Chinese patients; thus, a large cohort including different ethnic populations is needed to verify additional *OOEP* mutations. Third, the exact mechanism and potential signaling pathways involved in the effects of impaired embryo cleavage caused by gene mutations need further study using human samples or mouse models. Fourth, other potential factors that could contribute to the effect of SCMC‐related gene mutations on early embryo development should be clarified, including female age, male factors, and fertilization methods.

In conclusion, we found two compound heterozygous mutation variants in the *OOEP* gene and six compound heterozygous missense or frameshift variants in the *NLRP5* gene that might be related to human EEA. These mutations may contribute to the functional loss of OOEP and NLRP5 proteins to different extents, as shown in the in vitro experiments. We are the first to demonstrate that *OOEP* mutation modestly affects ZGA. Our study provides new evidence that SCMC‐associated gene mutations are responsible for human early embryonic development failure. Further studies are required to elucidate how mutations in *OOEP* and *NLRP5* affect human EEA.

## WEB RESOURCES

Online Mendelian Inheritance in Man (OMIM): http://www.ncbi.nlm.nih.gov/Omim

Genome Aggregation Database (GnomAD): http://gnomad.broadinstitute.org

Protein Variation Effect Analyzer (PROVEAN): http://provean.jcvi.org/index.php

Combined Annotation Dependent Depletion (CADD): https://cadd.gs.washington.edu/

## AUTHOR CONTRIBUTIONS

Xiaomei Tong, Jiamin Jin, Heng‐Yu Fan, Yin‐Li Zhang, and Songying Zhang conceived and designed the study. Xiaomei Tong and Jiamin Jin collected the clinical samples and medical records. Zhanhong Hu and Jiamin Jin performed the in vitro experiments. Yinyi Zhang conducted the single‐cell RNA‐seq experiment. Zhanhong Hu and Jiamin Jin analyzed the data. Xiaomei Tong and Jiamin Jin drafted the manuscript. Yin‐Li Zhang critically revised the manuscript. All authors reviewed and modified the article before approving it for publication.

## CONFLICT OF INTEREST

The authors declare no conflict of interest.

## Supporting information

Supporting information.

## Data Availability

The data that support the findings of this study are available on request from the corresponding author. The data are not publicly available due to privacy or ethical restrictions.
