# Supplementary material for: Mutations in OOEP and NLRP5 identified in infertile patients with early embryonic arrest
Source: Hum Mutat. 2022 Aug 30;43(12):1909–20. doi: 10.1002/humu.24448 (PMC10087254; doi:10.1002/humu.24448)
Supplement: Supplementary file 1 — Supporting information. [file HUMU-43-1909-s001.pdf]

**Supp. Table S1. Whole exome sequencing data of 5 patients with *OOEP* and *NLRP5* mutation.**

| Sample ID | *OOEP* | *NLRP5* | | | |
|---|---|---|---|---|---|
| | **Patient 1** | **Patient 2** | **Patient 3** | **Patient 4** | **Patient 5** |
| Raw reads (Mb) | 108.1 | 136.1 | 216.2 | 167.2 | 178.3 |
| Clean reads (Mb) | 107.1 | 134.3 | 215.3 | 166.6 | 177.7 |
| Accurate mapped bases (Mb) | 12778 | 15198 | 21530.77 | 16664.47 | 17778.98 |
| Accurate mapping rate (%) | 99.45 | 98.24 | 99.57 | 99.63 | 99.68 |
| Target size | 58231156 | 58231156 | 35735556 | 35735556 | 35735556 |
| Target covered size | 58082268 | 58091014 | 35592313 | 35574745 | 35571172 |
| Coverage rate (%) | 99.74 | 99.76 | 99.60% | 99.55% | 99.54% |
| Target mean depth | 136.93 | 170.07 | 305.86 | 210.47 | 254.67 |
| 4X coverage rate (%) | 99.62 | 99.65 | 99.34% | 98.93% | 98.89% |
| 10X coverage rate (%) | 99.25 | 99.40 | 99.16% | 98.81% | 98.68% |
| 20X coverage rate (%) | 98.17 | 98.72 | 99.00% | 98.67% | 98.48% |

**Supp. Table S2. Primers of *NLRP5* and *OOEP* mutation sites for Sanger analysis.**

| Mutation variant | Direction | Sequences (5'-3') | Target size (bp) |
|---|---|---|---|
| c.971T>A | Forward | CGTGATGACCAAATTCGCTGA | 667 |
| | Reverse | GTTCATGATCGCACGCAAC | |
| c.3341T>C | Forward | GGAGTATCGCTTGAATCCCA | 651 |
| | Reverse | CTGTATCTCCAGCATCCAG | |
| c.1575_1576delAG | Forward | GTCGTGTCTCCCCGTTACCTG | 689 |
| | Reverse | TCCAGTGGCCTCCTTACGTCT | |
| c.1830_1831delGT | Forward | ACGACCTCATGGTTCAAGGAC | 654 |
| | Reverse | ATAAAACTGCCTGGGGTACTCAC | |
| c.1202C>T | Forward | ACGGAGATCATGTCCCGACC | 633 |
| | Reverse | CTCCCCGAGTCCTTGAACCAT | |
| c.2378T>G | Forward | CACCGGGCTAGTCATGCAAA | 694 |
| | Reverse | GGATTACAGGCACGTACCAC | |
| c.109C>G | Forward | TTGCGAAAGCCGCCT | 477 |
| | Reverse | TCTGGAATTATGGCTCGGTCT | |
| c.110G>C | Forward | TTGCGAAAGCCGCCT | 477 |
| | Reverse | TCTGGAATTATGGCTCGGTCT | |

**Supp. Table S3. Primers of *NLRP5* and *OOEP* mutation sites for plasmid mutagenesis.**

| Mutation site | Direction | Sequences (5'-3') |
|---|---|---|
| c.971T>A | Forward | CTTCCTCCCCGTTAGAGAGAAGCAGCGGAAGAAGGAGAGCA |
| | Reverse | TGCTGTCCTTCTTCCGCTGCTTCTCTCTAACGGGGAGGAAG |
| c.3341T>C | Forward | TTCTGATTGCTGTGAGGCACCCTCCTTGGCCCTTTCCTGCA |
| | Reverse | TGCAGGAAAGGGCCAAGGAGGGTGCCTCACAGCAATCAGAA |
| c.1575_1576delAG | Forward | TGTCTCAATCTGGAGGAAAGTTGTCCTGAAGCGCTTCTGC |
| | Reverse | GCAGAAGCGCTTCAGGACAACTTTCCTCCAGATTGAGACA |
| c.1830_1831delGT | Forward | GAGCCAGCTCTCTGCCCTCTACGTTGAGAAGACAAAGAGG |
| | Reverse | CCTCTTTGTCTTCTCAACGTAGAGGGCAGAGAGCTGGCTC |
| c.1202C>T | Forward | GCTGAGGAAGGTCCTGCTCCTTGAGTCCTTCCTGATCGTCA |
| | Reverse | TGACGATCAGGAAGGACTCAAGGAGCAGGACCTTCCTCAGC |
| c.2378T>G | Forward | GGACCTGGGCAGCAGCATCCGGACAGAGCGGGCCATGAAGA |
| | Reverse | TCTTCATGGCCCGCTCTGTCCGGATGCTGCTGCCCAGGTCC |
| c.109C>G | Forward | CGCCGCCACAGATTCGCATCGGGCCCTGGTGGTTTCCGGTG |
| | Reverse | CACCGGAAACCACCAGGGCCCGATGCGAATCTGTGGCGGCG |
| c.110G>C | Forward | GCCGCCACAGATTCGCATCCCGCCCTGGTGGTTTCCGGTGC |
| | Reverse | GCACCGGAAACCACCAGGGCGGGATGCGAATCTGTGGCGGC |
